# Canal of Nuck hernia as an unusual cause of inguinal pain in women: a case report and laparoscopic management

**DOI:** 10.31744/einstein_journal/2026RC1823

**Published:** 2026-09-09

**Authors:** Claudio Zambotti, Giovanna Paes Scarin, Alexia Nascimento Arias, Luana Perrone Camilo, Gustavo Yano Callado, Eduardo Félix Martins Santana

**Affiliations:** 1 Hospital Israelita Albert Einstein São Paulo SP Brazil Hospital Israelita Albert Einstein, São Paulo, SP, Brazil.; 2 Universidade Santo Amaro Faculdade de Medicina São Paulo SP Brazil Faculdade de Medicina, Universidade Santo Amaro, São Paulo, SP, Brazil.; 3 Hospital Israelita Albert Einstein Faculdade Israelita de Ciências da Saúde Albert Einstein São Paulo SP Brazil Faculdade Israelita de Ciências da Saúde Albert Einstein, Hospital Israelita Albert Einstein, São Paulo, SP, Brazil.; 4 Universidade Federal de São Paulo Department of Obstetrics São Paulo SP Brazil Department of Obstetrics, Universidade Federal de São Paulo, São Paulo, SP, Brazil.

**Keywords:** Inguinal canal, Hernia, inguinal, Laparoscopy, Herniorrhaphy, Women's health services

## Abstract

We report a rare case of canal of Nuck herniation in an adult woman. It results from a persistent processus vaginalis, which can lead to the formation of hernias or hydroceles. A 34-year-old woman presented with acute right inguinal pain, and an ultrasound revealed a patent canal of Nuck containing a herniated intra-abdominal structure. The patient underwent laparoscopic transabdominal preperitoneal inguinal hernia repair, during which the hernia sac was successfully managed, and a polypropylene mesh was placed to reinforce the myopectineal orifice. This minimally invasive approach contributed to a rapid postoperative recovery, as evidenced by discharge on the first postoperative day. This case report underscores the importance of early imaging in diagnosing rare inguinal hernias in women and demonstrates the safety and effectiveness of laparoscopic transabdominal preperitoneal repair for canal of Nuck herniation.

## INTRODUCTION

Herniation of the canal of Nuck is a rare and frequently overlooked condition in adult women, typically diagnosed in infancy or early childhood. This anatomical remnant — a persistent processus vaginalis in females — may result in a hernia or hydrocele when it remains patent.^(1-3)^ While the condition is well-documented in pediatric populations, its presentation in adult women is both atypical and underreported in the literature, which often leads to diagnostic delays or misinterpretation.^(4)^

The hernia sac in such cases may contain omentum, bowel loops, or even reproductive structures such as the ovary or uterus. In adult women, right-sided hernias involving the appendix are particularly rare and pose diagnostic challenges, especially when clinical signs are subtle or mimic more common causes of inguinal symptoms. This scenario may raise suspicion for an Amyand hernia, defined as the presence of the vermiform appendix within an inguinal hernia sac — a rare but relevant differential diagnosis in women experiencing groin pain. Early and accurate imaging evaluation plays a pivotal role in avoiding unnecessary interventions and guiding appropriate surgical planning.^(1,2,5)^

This report describes a rare case of a right-sided canal of Nuck hernia in a previously healthy adult woman, with herniation of the vermiform appendix. This case not only reinforces the importance of imaging assessment but also highlights the safety and effectiveness of the laparoscopic TAPP approach as a definitive repair technique in adult women.

## CASE REPORT

A 34-year-old previously healthy woman was admitted to the emergency department of a tertiary private hospital in São Paulo, Brazil, with acute right inguinal pain. Soft tissue ultrasonography performed on admission revealed a protrusion of intra-abdominal contents through the right inguinal canal, consistent with a patent canal of Nuck (Figure 1). The protruded material included a vermiform-appearing structure suggestive of the vermiform appendix, measuring approximately 0.5cm in diameter, with preserved vascularity on Doppler evaluation. Although ultrasound findings were suggestive of a right-sided canal of Nuck hernia containing intra-abdominal contents — most likely the appendix — definitive identification was not possible at this stage. No abnormalities were observed in the skin, subcutaneous tissue, or muscle planes, and no masses or fluid collections were detected. Importantly, there were no sonographic signs of ischemia or inflammation.

**Figure 1:**
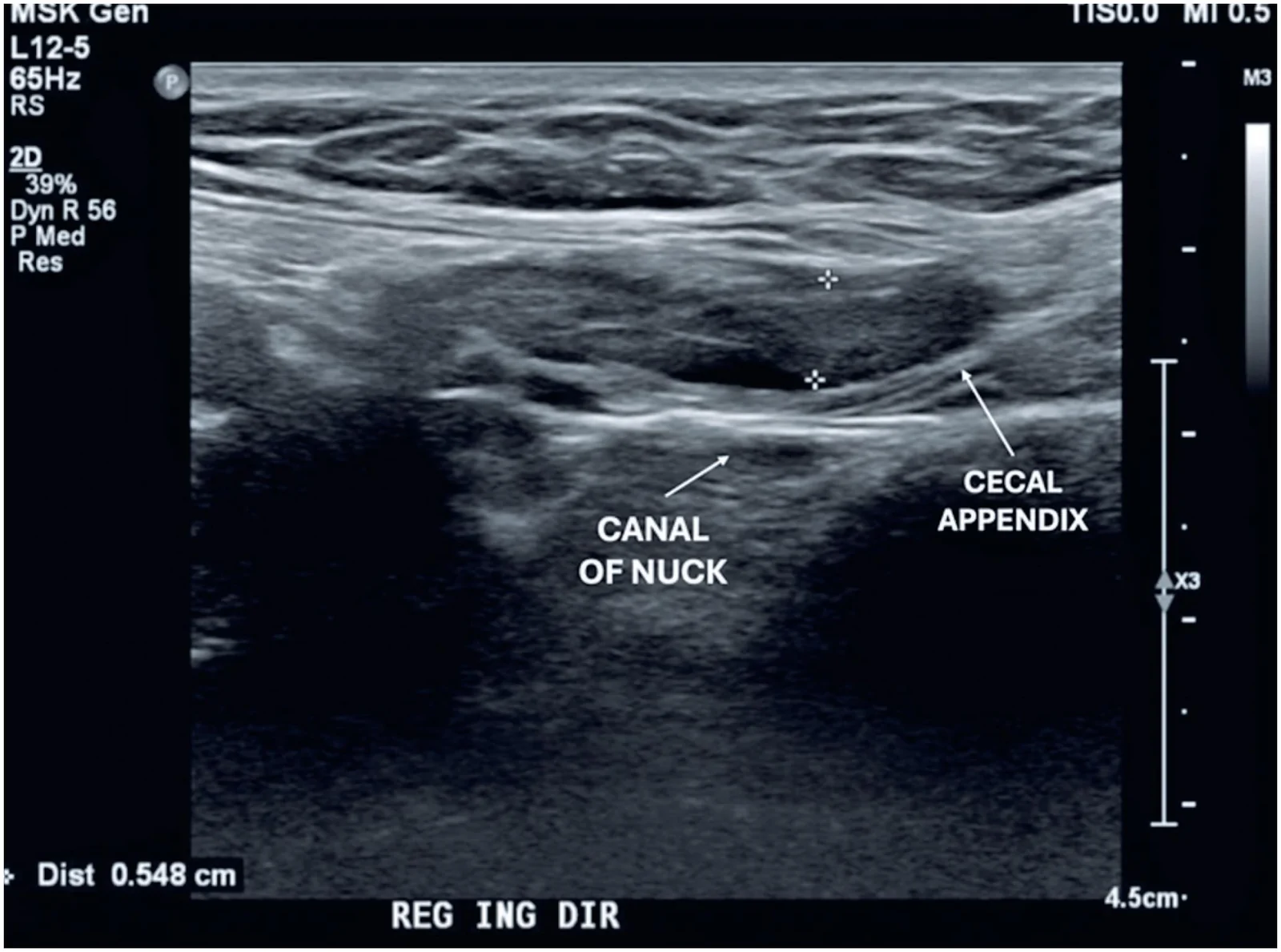
Ultrasonographic Identification of the Patent Canal of Nuck with Appendix Herniation. Soft tissue ultrasonography of the right inguinal region performed at admission revealed a tubular protrusion of intra-abdominal content through the inguinal canal, consistent with a patent canal of Nuck. Notably, a vermiform structure within the canal, suggestive of the vermiformappendix, measured 0.548 cm in diameter

Analgesia was administered, leading to symptom improvement, and the patient was discharged from the emergency department with symptomatic management and referred for outpatient evaluation with a general surgeon. During the specialist consultation, a lower abdominal computed tomography (CT) scan was requested and performed with intravenous contrast during a Valsalva maneuver to assess the inguinal region. The scan revealed an intact abdominal wall with no visible herniation during Valsalva, and both inguinal canals appeared normal in caliber, without focal lesions. However, asymmetric thickening of the right round ligament and the adjacent inguinal canal was noted. The urinary bladder demonstrated minimal filling with homogeneous content, intestinal loops appeared unremarkable, and no lymphadenopathy was identified. Lastly, a small volume of free pelvic fluid was observed.

### Surgical management

The patient subsequently underwent right laparoscopic inguinal hernia repair via the transabdominal preperitoneal (TAPP) approach. Intraoperative exploration and dissection of the canal of Nuck revealed a hernia sac containing the vermiform appendix, confirming the preoperative ultrasonographic findings (Figure 2). The procedure was uneventful, and the patient was discharged on postoperative day 1 without complications.

**Figure 2:**
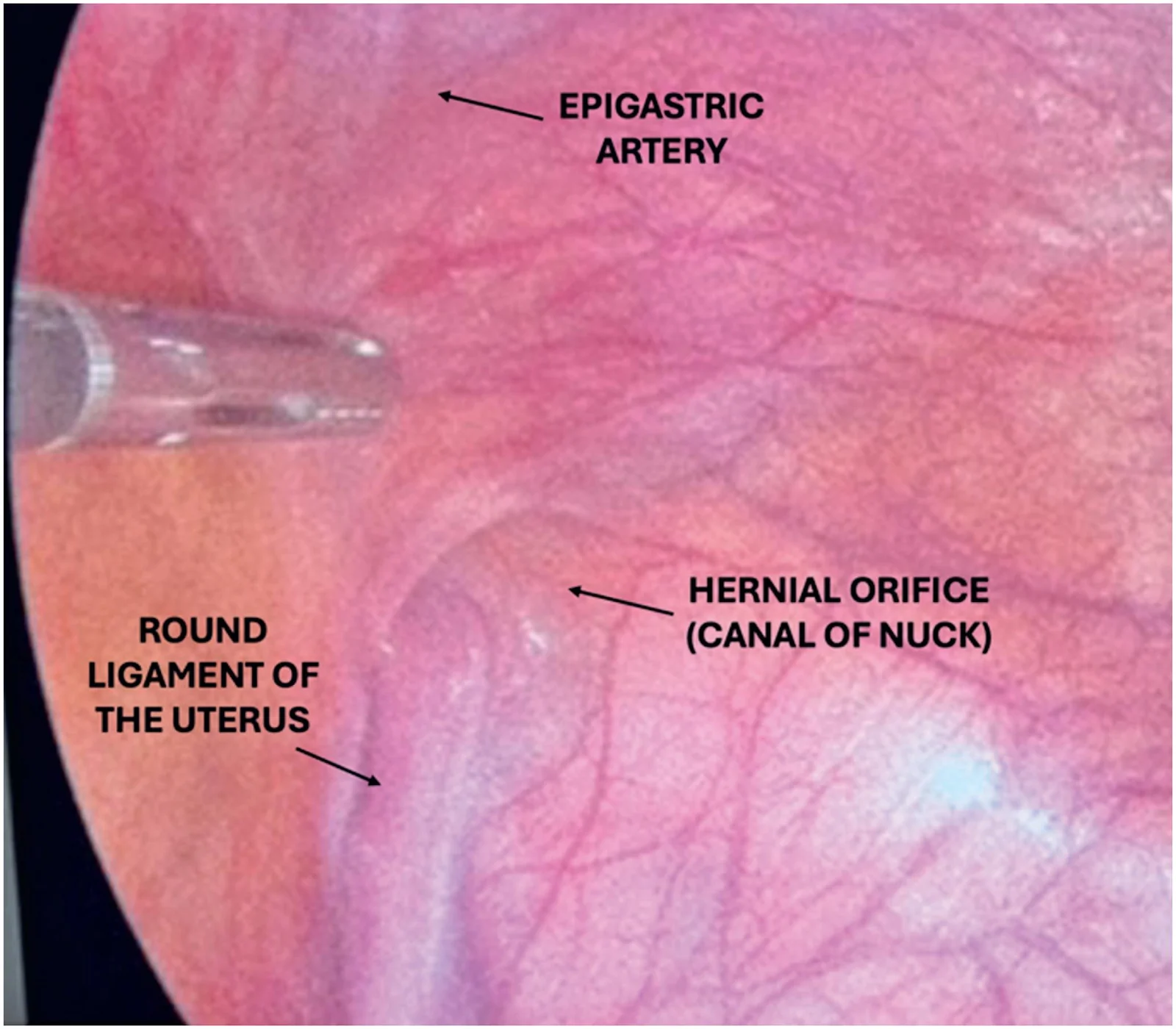
Laparoscopic View of the Hernia Sac Containing the Appendix within the Canal of Nuck. Intraoperative laparoscopic exploration and dissection of the canal of Nuck revealed a hernia sac containing the vermiform appendix appendix. The appendix appeared intact and was identified as the sole content of the herniated sac

Laparoscopic hernia repair in this context involved the placement of a tension-free polypropylene mesh covering the myopectineal orifice. This minimally invasive technique offers several advantages, including enhanced anatomical visualization, faster recovery, reduced postoperative pain, and a lower risk of intraperitoneal visceral injury. Access to the peritoneal cavity was achieved through an umbilical incision using a Veress needle to create pneumoperitoneum, followed by insertion of one 11mm trocar and two 5mm trocars bilaterally.

Intraoperative inspection confirmed the presence of a right-sided indirect inguinal hernia. The peritoneal flap was opened using scissors and electrocautery to expose the inguinal region and its anatomical structures. Dissection of the canal of Nuck was performed to identify any associated structures, such as cystic formations, which were not observed in this case.

The repair was completed by reinforcing the posterior wall with a polypropylene mesh, ensuring coverage of all areas at risk for recurrence or future herniation. Mesh fixation was achieved using absorbable tacks, followed by closure of the peritoneal flap with a continuous barbed suture. Pneumoperitoneum was released, and trocar incisions were closed with absorbable intradermal sutures and skin adhesive.

The case report was approved by the Research Ethics Committee of the *Hospital Israelita Albert Einstein*, CAAE: 96600426.8.0000.0071; # 8.312.571.

## DISCUSSION

This case underscores two pivotal aspects that emphasize its clinical relevance: the rarity of a patent canal of Nuck hernia in an adult woman and the effectiveness of its surgical management through a minimally invasive TAPP approach. Despite being well recognized in pediatric populations, this condition is uncommon and frequently underdiagnosed in adults. Moreover, preoperative identification of herniated content suggestive of the appendix, combined with an elective laparoscopic repair, highlights the value of early imaging and minimally invasive surgical techniques in optimizing outcomes. Although imaging only raised the suspicion of an appendix-containing hernia (potential Amyand-type hernia), intraoperative findings confirmed the presence of the vermiform appendix within the hernia sac.

The patent canal of Nuck in adult women is a rare but well-documented condition in the medical literature.^(6,7)^ Hydroceles of the canal of Nuck are described as uncommon causes of inguinal swelling in females and are frequently associated with pain.^(8)^ The rarity of this entity, along with its nonspecific clinical presentation, contributes to diagnostic challenges, as it can be easily mistaken for other causes of inguinal pain or palpable masses. Ultrasonography is commonly employed as the first-line imaging modality for evaluating these conditions.^(9)^ Additionally, the persistence of the canal of Nuck is associated with the development of indirect inguinal hernias in women, analogous to inguinoscrotal hernias in men, in which intra-abdominal contents protrude through the patent peritoneal extension.

Diagnosis of a canal of Nuck hernia relies primarily on imaging. Ultrasonography is usually the first-line modality due to its accessibility, safety, and ability to differentiate between solid and cystic structures. It allows identification of herniated contents, evaluation of vascular flow to exclude strangulation, and differentiation from other inguinal pathologies such as lymphadenopathy, lipomas, and abscesses. Computed tomography, particularly when performed with maneuvers such as Valsalva, adds anatomical detail and can be useful in preoperative planning. Magnetic resonance imaging may be employed in complex or unclear cases. It is relevant to note that the absence of a detectable hernia on CT, even when performed during a Valsalva maneuver, does not definitively exclude the diagnosis of a canal of Nuck hernia. This is particularly true in cases where the herniation is small, reducible, or intermittent. Therefore, ultrasound may offer greater sensitivity in certain clinical scenarios, especially when correlated with physical examination findings.

Early diagnosis is critical to prevent complications such as incarceration, strangulation, or torsion of herniated structures, particularly when reproductive organs or the appendix are involved. Delayed identification increases the risk of acute abdomen, ischemia, and the need for emergency surgical intervention. In certain instances, especially those involving the ovary, irreversible damage may occur, potentially compromising fertility. Therefore, prompt recognition and elective repair are recommended, even in mildly symptomatic patients.^(1-3)^

Open surgical repair remains a traditional approach, especially in emergency settings or when laparoscopy is contraindicated.^(10,11)^ It offers direct access to the inguinal canal, facilitates identification of the hernia sac, allows for reduction of herniated contents, and enables definitive closure of the defect, often reinforced with mesh.^(12)^ Although effective, open repair may be associated with a longer recovery period, greater postoperative discomfort, and higher rates of complications, including wound infections and nerve injury, when compared to minimally invasive techniques.

The TAPP approach, as employed in this case, confers several advantages. It provides enhanced visualization of intra-abdominal structures, enables meticulous dissection of the hernia sac, and allows for bilateral exploration through the same incisions. Transabdominal preperitoneal is associated with reduced postoperative pain, expedited recovery, and lower rates of surgical site complications.^(13)^ Additionally, the placement of a large preperitoneal mesh facilitates a tension-free repair and minimizes the risk of recurrence. For rare entities such as canal of Nuck hernias, laparoscopy permits a thorough assessment of pelvic and inguinal anatomy, which may aid in detecting subtle or atypical findings.^(14,15)^

## CONCLUSION

This case illustrates that appropriate imaging, combined with a minimally invasive laparoscopic approach, can lead to favorable outcomes in women with a canal of Nuck hernia, including early recovery and minimal postoperative complications.

## Data Availability

The underlying content is contained within the manuscript.
